# Critical Assessment of the Recommended Alert Limit Curves for Occupational Heat Exposure

**DOI:** 10.1002/ajim.70022

**Published:** 2025-09-07

**Authors:** Hayden W. Hess, M. Jo Hite, Molly E. Heikkinen, Macie L. Tarr, Erica Tourula, Blair D. Johnson, David Hostler, Zachary J. Schlader

**Affiliations:** ^1^ Department of Kinesiology Indiana University School of Public Health Bloomington Indiana USA; ^2^ Department of Exercise and Nutrition Sciences, Center for Research and Education in Special Environments University at Buffalo Buffalo New York USA

**Keywords:** heat strain, heat stress, hydration, recommendations

## Abstract

**Background:**

Occupational heat stress recommendations aim to achieve thermal equilibrium and keep core temperature (*T*_c_) below 38.0°C. We assessed the recommended alert limit curves when: (1) work–rest ratios are adjusted based on wet‐bulb globe temperature (WBGT) at a fixed rate of metabolic heat production (*H*_prod_) and (2) *H*_prod_ is adjusted based on WBGT at a fixed work–rest ratio. We tested the hypothesis that adhering to occupational heat stress recommendations results in thermal equilibrium and prevents *T*_C_ from exceeding 38.0°C.

**Methods:**

Unacclimated adults completed 4‐hour exposures at a fixed WBGT, *H*_prod_, and work–rest ratio. There were six iterations of compliant trials (*n* = 70 observations; A: 24.1°C, 431 W, 60:0 min; B: 26.6°C, 461 W, 45:15 min; C: 28.4°C, 462 W, 30:30 min; D: 29.7, 453 W, 15:45 min; E: 27.3°C, 412 W, 30:30 min; F: 31.6°C, 290 W, 30:30 min) and two iterations of noncompliant trials (*n* = 24 observations; G: 31.6°C, 413 W, 30:30 min; H: 36.1°C, 453 W, 15:45 min).

**Results:**

Mean and peak *T*_C_ across the compliant trials were 37.6°C ± 0.3°C and 37.9°C ± 0.3°C. Thus, 65/70 (93%) and 44/70 (63%) trials did not exceed a mean or peak core *T*_C_ of 38.0°C. Mean and peak *T*_C_ across the noncompliant trials exceeded 38.0°C in all trials. The rate of heat gain differed between compliant and noncompliant trials (0.08°C ± 0.07°C/h vs. 0.41°C ± 0.34°C/h; *p* < 0.0001) but on average thermal equilibrium was < 0.1°C/h in the compliant trials.

**Conclusion:**

Compliance with the occupational heat stress recommendations resulted in thermal equilibrium and mitigated the development of excessive heat strain.

**Trial Registration:**

ClinicalTrials.gov: NCT04767347.

## 1 Introduction

Occupational heat exposure increases the risk of heat‐related injury and/or illness [1, 2]. This occupational health problem is likely to worsen due to rising global temperatures resulting in more intense and frequent exposure to extreme heat [3]. The burden of excess heat stress among laborers results in both economic (e.g., labor loss, reduction in productivity) and short‐ and long‐term health consequences [1, 4]. Therefore it is imperative to establish viable methods for limiting heat stress in this population. As such, federal and nonfederal agencies, such as World Health Organization (WHO), American Conference of Governmental Industrial Hygienists, and the National Institute for Occupational Safety and Health (NIOSH), issue exposure limit recommendations to mitigate the risk of developing hyperthermia and heat‐related injury and illness.

**Figure 1:**
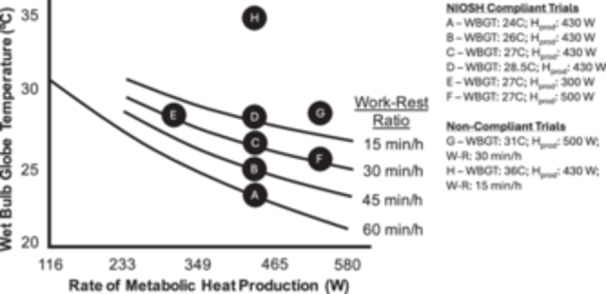
Study design and experimental trials on the basis of the NIOSH recommended alert limit curves [5]. Participants walked on a treadmill at NIOSH‐compliant work intensities (i.e., metabolic heat production) as a function of wet‐bulb globe temperature (WBGT) and work–rest ratio.

Regardless of agency, these exposure limits aim to ensure that thermal equilibrium (i.e., an increase in core temperature ≤ 0.1°C/h) can be achieved and the average worker's core temperature does not exceed 38.0°C in unacclimatized workers or 38.5°C in acclimatized workers [5, 6]. The 38.0°C core temperature threshold originated from a WHO technical report [7] which stated that it is inadvisable for deep core body temperature to exceed 38.0°C during prolonged, heavy daily work. This 38.0°C threshold is supported by conjecture that when work is prescribed to ensure core temperature does not exceed 38.0°C, then ≥ 95% of a population of workers will not exceed a core body temperature of 39.2°C (i.e., excessive heat strain) or higher, thereby avoiding heat‐related sequelae [8, 9]. In the United States, NIOSH issues recommended alert limits (RALs) or recommended exposure limits (RELs) for unacclimatized and acclimatized workers, respectively, to achieve thermal equilibrium and, ultimately, reduce the likelihood of core temperature exceeding 38.0°C [5]. Both RALs and RELs establish wet‐bulb globe temperature (WBGT) limits as a function of time‐weighted average metabolic heat production (see NIOSH [5, eq. 8.1]). However, there are challenges in determining the management of total work (as a function of intensity) and when to issue work–rest guidance. NIOSH published visualization of these curves as a function of WBGT and metabolic heat production to aid in determining appropriate work–rest ratios (see NIOSH [5, Figure 8‐1 [RAL] and Figure 8‐1 [REL]]). However whether — utilizing the recommended work–rest ratios for a given time‐weighted average (i.e., at rest and during work) — metabolic heat production results in thermal equilibrium and prevention of the average worker's core temperature from exceeding 38.0°C has yet to be empirically examined. Furthermore, as indicated by Bernard et al. [10], there are unintentional discrepancies between the equations used to prescribe RALs and the figure that visually depicts these curves. The authors state that the equations are taken to be the intended limit [10]. However, this discrepancy has not been critically assessed.

With this background, the purpose of the present paper was to: (1) examine the effectiveness of using the NIOSH curves to establish exposure limits to achieve thermal equilibrium and prevent mean and/or peak core body temperature from exceeding 38.0°C; and (2) compare the core temperature responses during compliant and noncompliant trials (including the former Ceiling Limit [11]). We hypothesized that adherence to the NIOSH curves would result in thermal equilibrium, prevent mean core temperature from exceeding 38.0°C, and maintain a core temperature lower than in noncompliant trials. An exploratory analysis was also completed to determine the magnitude of the discrepancy between the figure and the equations and its influence on core temperature responses during a simulated half workday in the heat.

## 2 Methods

### 2.1 Participants

A total of 24 healthy adult nonsmokers (Table 1) completed 3–5 experimental trials each. All subjects were free of chronic disease, were not heat acclimatized [12], and regularly engaged in physical activity as reported via the International Physical Activity Questionnaire [13]. Women were not pregnant, which was confirmed via urine pregnancy test prior to each visit, self‐reported to be normally menstruating, and had no diagnosis of a menstrual cycle disorder. Women were tested at any point during their menstrual cycle because in real‐world settings women work across their entire menstrual cycle. The data presented here were combined from two independent studies in which a portion of the data were previously published in manuscripts that tested unique hypotheses [14, 15, 16, 17]. The studies were approved by the Institutional Review Board at Indiana University (IRB# 1902420140), conformed to the Declaration of Helsinki, and were registered at clinicaltrials.gov (NCT04767347).

**Table 1:** Subject characteristics.

Subjects, *n*	Total: 24 Men: 11 Women: 13
Age, yr	28 ± 6 [21, 38]
Height, cm	176 ± 8 [165, 190]
Weight, kg	75 ± 13 [59, 93]
Body mass index, kg/m^2^	24 ± 3 [18, 29]
Body surface area, m^2^	1.9 ± 0.2 [1.7, 2.2]
Heart rate, bpm	65 ± 12 [51, 91]
Systolic blood pressure, mmHg	120 ± 13 [97, 135]
Diastolic blood pressure, mmHg	73± 8 [60, 86]

*Note:* Values are means ± SD [min, max].

### 2.2 Experimental Protocol

The study design and experimental protocol have been described in detail previously [14, 15, 16, 17] (Figure 1). The first visit involved screening. Subjects then completed up to five experimental trials separated by at least 3 days (Trials A–D and Trial H from Hess et al. [14, 15, 17] and Trials E–G from Hess et al. [16]). All subjects completed the studies in 6–12 weeks. Experimental trial characteristics are presented in detail in Table 2. In all trials, subjects completed a 4‐h workday simulation. In NIOSH‐compliant trials (Trials A–F), work–rest ratios (work:rest min/h) were prescribed as a function of WBGT and *H*_prod_. Noncompliant trials were conducted at a work intensity too high for a given WBGT and work–rest ratio (Trial G), or in environmental conditions that reflected what was previously known as the ceiling limit, defined as the threshold WBGT that NIOSH would stipulate that work would be contraindicated [11] (Trial H).

**Table 2:** Comparison of compliant vs. noncompliant trials.

Parameter	Compliant		Noncompliant
Number of observations	70		24
WBGT (°C)			
A (*n* = 11) B (*n* = 11) C (*n* = 12) D (*n* = 12) E (*n* = 12) F (*n* = 12)	24.1 ± 0.3 26.6 ± 0.2 28.4 ± 0.2 29.7 ± 1.6 31.5 ± 0.5 27.0 ± 0.1	G (*n* = 12) H (*n* = 12)	31.6 ± 0.1 36.1 ± 0.3
*H*_prod_ (W)			
A B C D E F	431 ± 101 461 ± 106 462 ± 91 453 ± 105 299 ± 55 410 ± 55	G H	413 ± 63 453 ± 113
TWA‐*H*_prod_ (W)			
A B C D E F	431 ± 101 373 ± 80 294 ± 62 215 ± 39 259 ± 34 195 ± 50	G H	256 ± 31 200 ± 45
TWA‐WBGT (°C)			
A B C D E F	22.9 ± 1.4 23.8 ± 1.4 25.2 ± 1.3 27.1 ± 1.1 25.9 ± 0.8 27.8 ± 1.5	G H	26.0 ± 0.8 27.6 ± 1.2
ΔRAL (°C)			
A B C D E F	1.2 ± 1.3 2.8 ± 1.3 3.2 ± 1.2 2.6 ± 2.1 1.4 ± 1.2 3.8 ± 1.4	G H	5.7 ± 0.8 8.5 ± 1.3

*Note:* Environmental conditions, metabolic heat production, time‐weighted average metabolic heat production, and recommended alert limits for unacclimatized subjects during NIOSH compliant (A–F) and noncompliant (G and H) trials. Values are means ± SD.

Abbreviations: *H*_prod_, metabolic heat production; RAL, recommended alert limit; TWA, time‐weighted average; WBGT, wet‐bulb globe temperature.

### 2.3 Data and Statistical Analyses

#### 2.3.1 Core Temperature

Core temperature was measured every 15 min during the 4‐h exposures by a rectal temperature probe inserted ~10 cm beyond the anal sphincter (*n* = 15) or by telemetry pill (HQ Inc.) swallowed ~6–8 h before each experimental trial (*n* = 9). Peak core temperature (i.e., the highest recorded core temperature within each 4‐h exposure) and mean core temperature (i.e., the mean of all core temperature recordings within each trial) are reported. The rationale of including both mean and peak core temperature is that heat‐related organ injury is likely contributed to by total heat load across a given workday (e.g., time above a given core temperature) or the maximum core temperature experienced [18] whereas peak core temperature is acknowledged as an important consideration in severe heat‐related illness (e.g., heat stroke [19]). Individual core temperature responses from Hours 2–4 were fit using linear regression and the resulting slope equation was utilized to quantify thermal equilibrium within a given trial. Based on previous work [10], we defined thermal equilibrium as a slope of ≤ 0.1°C/h over this time period. To determine if the rate of the increase in core temperature was different between compliant trials and noncompliant trials, the slopes of the linear regression (°C/h) were analyzed utilizing a one‐way linear mixed model (compliant trials only) or using unpaired *t*‐tests when comparing noncompliant Trials G and H or when comparing all compliant versus all noncompliant trials.

#### 2.3.2 Hydration

Metrics of hydration and fluid balance were measured in each experimental trial. Percentage changes in body mass were calculated from nude body mass measured pre‐ and postexposure. Total ad libitum volume consumed across each 4‐h exposure was measured. Average hourly sweat rate was calculated ([preexposure nude mass − postexposure mass − total fluid volume + urine output]/4). All metrics were analyzed utilizing a one‐way linear mixed model when comparing between the compliant trials or using unpaired *t*‐tests when directly comparing noncompliant Trials G and H or when comparing compliant versus noncompliant trials.

#### 2.3.3 Recommended Alert Limit (RAL)

RALs (i.e., time weighted average WBGT [TWA‐WBGT]) were quantified using the equation for unacclimatized workers (RAL = 59.9 − 14.1 log_10_ TWA‐M). The time‐weighted average metabolic heat production (TWA‐M) was calculated using the *H*_prod_ during exercise multiplied by the proportion of time exercise plus resting *H*_prod_ multiplied by the proportion of time resting (example: 430 W * 0.5 + 100 W * 0.5). *H*_prod_ was calculated using indirect calorimetry [20] by measuring the rates of oxygen uptake and carbon dioxide production via a metabolic cart over a 5‐min period before (i.e., at rest) and during the first 5 min of treadmill walking in each hour. The difference between the observed WBGT and the RAL was calculated as WBGT[observed] − WBGT[calculated] (i.e., ΔRAL). This permitted the determination of the magnitude of discrepancy between the RAL curves (NIOSH [5, Figure 8‐1]) and the RAL equation and its influence on core temperature responses. Two analyses were carried out. First, multiple linear regression was used to examine the relations between ΔRAL and trial on mean and peak core temperature and core temperature slope. This permitted examination of the discrepancy between the NIOSH [5] RAL curves versus the RAL equations and its influence on mean and peak core temperature and on core temperature slope. Second, multiple logistic regression analyses were completed to determine the independent influence of ΔRAL and trial on mean and peak core temperature and on the core temperature slope, where a mean and peak core temperature of ≥ 38.0°C and a core temperature slope of > 0.1°C/h were considered as “excessive heat strain” (e.g., < 38°C = 0; > 38°C = 1) or “uncompensable events” (e.g., < 0.1°C/h = 0; > 0.1°C/h = 1). Including both trial and ΔRAL in these models also permitted assessment of the independent effect of interindividual differences in *H*_prod_ on core temperature outcomes for a given WBGT exposure.

Data were analyzed with GraphPad Prism software (Version 10.2.0). All data are presented as individual or summarized data using mean ± SD. *A priori* statistical significance was set at *p* ≤ 0.05. In all instances, when a significant effect was identified, multiple comparisons were carried out using Sidak's test, which corrects for multiple comparisons. Actual *p* values are reported where possible. In instances referring to more than one pairwise comparison, the highest (for groups of comparisons where *p* ≤ 0.05) or lowest (for groups of comparisons where *p* ≥ 0.05) *p* values are reported using ≤ or ≥ symbols, respectively.

## 3 Results

**Figure 2:**
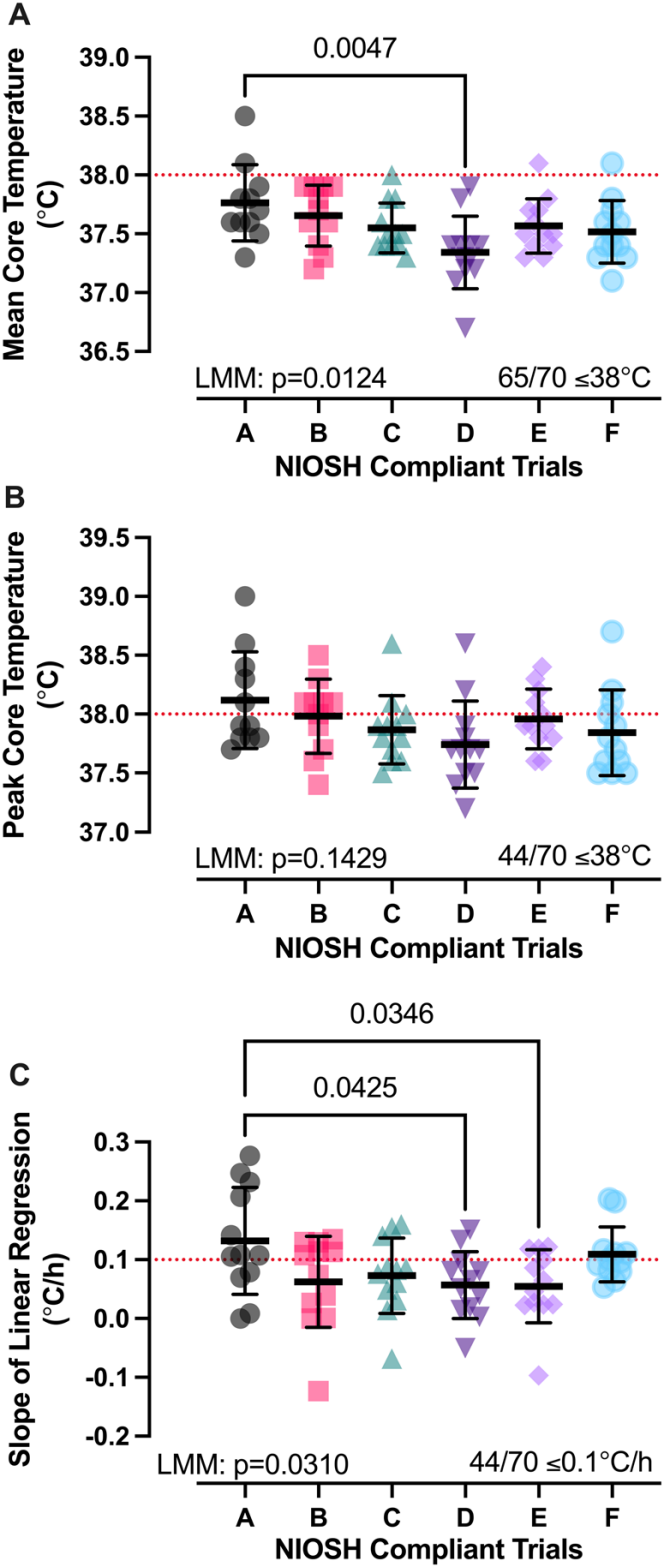
(A–C) Mean and peak core temperature responses and core temperature slope for the NIOSH compliant trials. Data are presented as individual values and as mean ± SD. Data were analyzed using a one‐way linear mixed model and exact *p* values reported.

**Figure 3:**
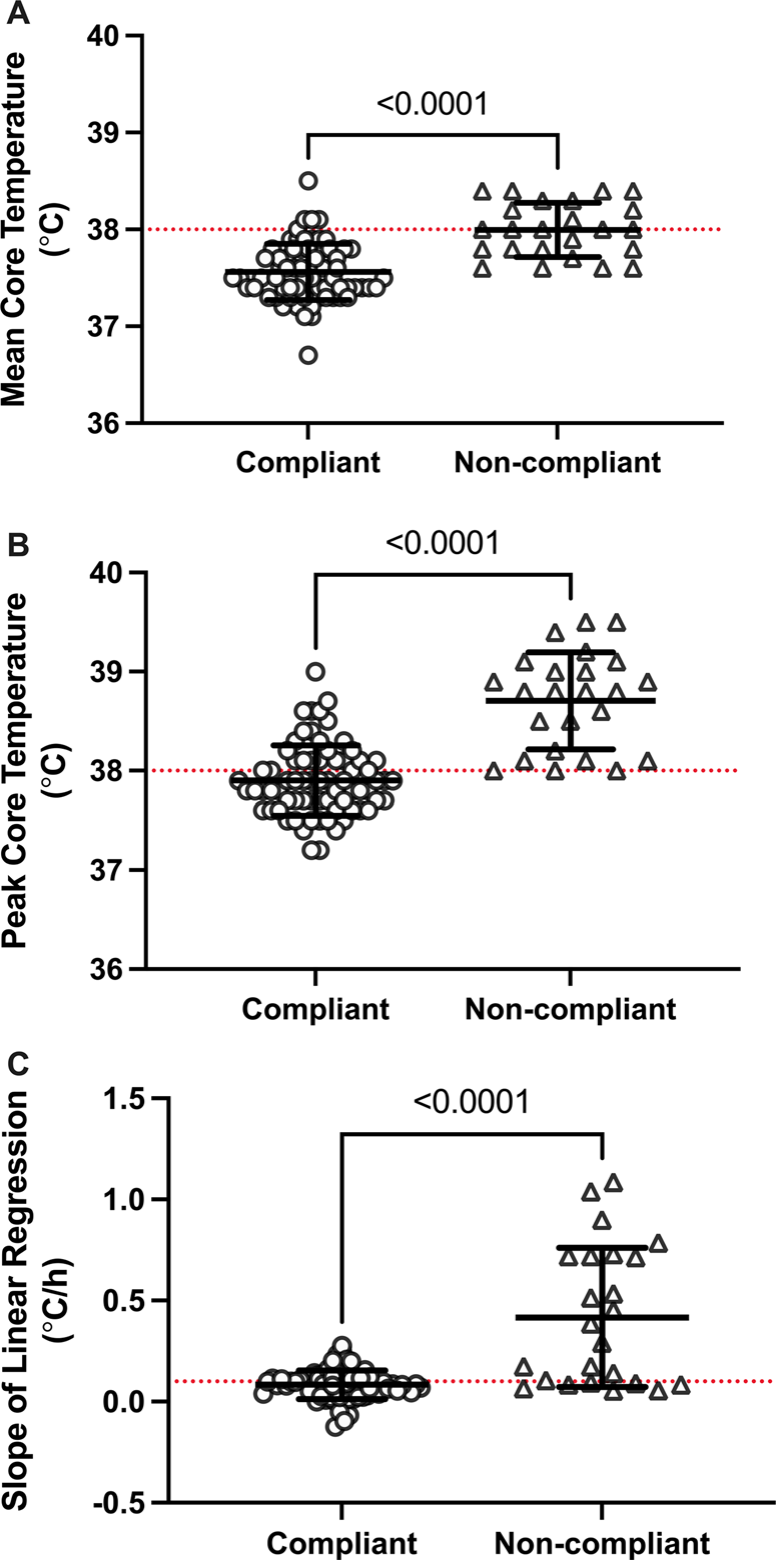
(A–C) Comparison of mean and peak core temperature responses and core temperature slope between NIOSH compliant and noncompliant trials. Data are presented as individual values and as mean ± SD. Data were analyzed using an unpaired *t*‐test and exact *p* values are reported. *n* = 70 (compliant trials) and *n* = 24 (noncompliant trials).

Experimental trial characteristics are presented in Table 2. Mean and peak core temperature responses and core temperature slope for the NIOSH compliant trials are presented in Figure 2A–C. Comparisons between NIOSH compliant and noncompliant trials for mean and peak core temperature responses and core temperature slope are presented in Figure 3A–C. The slope of the linear regression (Figure 2C) was different between compliant trials (*p* = 0.0310). Trial A (0.13 ± 0.09°C/h) was different than Trial D (0.06 ± 0.06°C/h; *p* = 0.0425) and Trial E (0.05 ± 0.06°C/h; *p* = 0.0346). The slope of the linear regression (Figure 3C) was different between compliant (0.08 ± 0.07°C/h) and noncompliant trials (0.41 ± 0.34°C/h; *p* < 0.0001).

**Figure 4:**
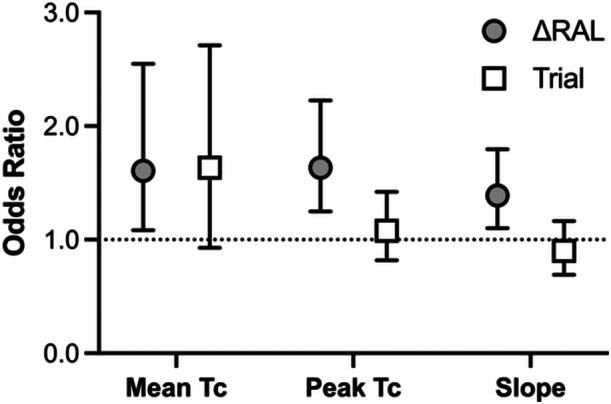
Odds ratio estimates and 95% confidence intervals from the multiple logistic regression analyses with independent variables of ΔRAL and trial, and dependent variables of mean and peak core temperature, and the core temperature slope where a mean and peak core temperature of ≥ 38.0°C, and a core temperature slope of > 0.1°C/h were considered “excessive heat strain” (e.g., < 38°C = 0; > 38°C = 1) or “uncompensable events” (e.g., < 0.1°C/h = 0; > 0.1°C/h = 1).

The ΔRAL (Table 2) was different between compliant trials (*p* = 0.0002) and between noncompliant trials (*p* < 0.0001). Multiple linear and logistic regression analyses are presented in Tables 3 and 4. To summarize, ΔRAL, and not trial, was consistently identified as a significant independent predictor of mean and peak core temperature and core temperature slope (Table 3). Similarly, ΔRAL, and not trial, were identified as significant independent predictors of when mean and peak core temperature exceed 38.0°C and when the core temperature slope exceeds 0.1°C/h (Table 4). As a result, for every 1°C increase in ΔRAL the odds of mean and peak core temperature exceeding 38.0°C increase by 61% and 63%, while the odds that the core temperature slope exceeds 0.1°C/h increase by ~39% (Figure 4). Finally, metrics of hydration and fluid balance are presented in Table 5.

**Table 3:** Multiple linear regression.

Dependent variable	Parameter	Variable	Estimate	Standard error	*p* value
Mean core temperature (°C)					
	*β*0	Intercept	37.42	0.07	< 0.0001
	*β*1	ΔRAL	0.07	0.02	< 0.0001
	*β*2	Trial	−0.004	0.02	0.8340
*R* ^2^ = 0.2831		Adjusted *R* ^2^ = 0.2672		AIC = −223.1	
Peak core temperature (°C)					
	*β*0	Intercept	37.56	0.09	< 0.0001
	*β*1	ΔRAL	0.11	0.02	< 0.0001
	*β*2	Trial	0.03	0.02	0.3133
*R* ^2^ = 0.4279		Adjusted *R* ^2^ = 0.4155		AIC = −168.8	
Slope (°C/h)					
	*β*0	Intercept	−0.08	0.04	0.0447
	*β*1	ΔRAL	0.05	0.01	< 0.0001
	*β*2	Trial	0.01	0.01	0.1900
*R* ^2^ = 0.4363		Adjusted *R* ^2^ = 0.4240		AIC = −323.7	

*Note:* Data from both compliant and noncompliant trials were analyzed using multiple linear regression when mean core temperature, peak core temperature, and slope are the dependent outcome variables (e.g., Mean core temperature (°C)~Intercept [*β*0] + ΔRAL [*β*1] + Trial [*β*2]).

Abbreviation: AIC, Akaike information criterion.

**Table 4:** Multiple logistic regression.

Dependent variable	Parameter	Variable	Estimate	95% CI	*p* value
Mean core temperature (°C)					
	*β*0	Intercept	−5.821	−8.779 to −3.749	< 0.001
	*β*1	ΔRAL	0.474	0.079 to 0.936	0.0178
	*β*2	Trial	0.394	−0.075 to 0.997	0.1033
AIC = 62.91					
Peak core temperature (°C)					
	*β*0	Intercept	−1.984	−3.186 to −0.911	0.0002
	*β*1	ΔRAL	0.491	0.222 to 0.799	0.0002
	*β*2	Trial	0.076	−0.199 to 0.351	0.5835
AIC = 109.0					
Slope (°C/h)					
	*β*0	Intercept	−0.8937	−1.874 to 0.042	0.0614
	*β*1	ΔRAL	0.3277	0.096 to 0.5850	0.0049
	*β*2	Trial	−0.1044	−0.369 to 0.151	0.4258
AIC = 127.0					

*Note:* Data from both compliant and noncompliant trials were analyzed using multiple logistic regression when mean core temperature, peak core temperature, and slope are the dependent outcome variables (e.g., Mean core temperature [< 38°C = 0; > 38°C = 1]~Intercept [*β*0] + ΔRAL [*β*1] + Trial [*β*2]).

Abbreviations: CI, confidence interval; AIC, Akaike information criterion.

**Table 5:** Hydration status and fluid balance.

Compliant trials (*n* = 12/trial)								Noncompliant trials (*n* = 12/trial)			Combined trials		
Trial/parameter	A	B	C	D	E	F	ANOVA	G	H	*t*‐test	Compliant trials combined (*n* = 72)	Noncompliant trials combined (*n* = 24)	*t*‐test
Preexposure body mass (kg)	75.9 ± 12.7	75.5 ± 12.7	75.5 ± 12.9	76.1 ± 12.6	73.9 ± 11.7	74.0 ± 11.9	*p* = 0.9963	74.1 ± 11.9	75.6 ± 12.6	*p* = 0.7671	75.1 ± 12.0	74.9 ± 12.1	*p* = 0.9439
Postexposure body mass (kg)	75.3 ± 12.7	75.1 ± 12.6	75.2 ± 12.6	75.8 ± 12.6	73.8 ± 11.6	74.1 ± 12.0	*p* = 0.9987	74.1 ± 11.9	75.5 ± 12.9	*p* = 0.7849	74.9 ± 11.9	74.8 ± 12.1	*p* = 0.9718
ΔBody mass (%)	−0.8 ± 0.9	−0.6 ± 0.6	−0.4 ± 0.6	−0.3 ± 0.6	−0.1 ± 0.8	0.1 ± 1.0	*p* = 0.0666	0.0 ± 1.2	−0.2 ± 1.7	*p* = 0.7423	−0.3 ± 0.8	−0.1 ± 1.5	*p* = 0.4061
Ad libitum fluid intake (L)	1.7 ± 0.8	1.7 ± 0.7	1.6 ± 0.5	1.4 ± 0.6	1.8 ± 0.9	1.7 ± 0.9	*p* = 0.4071	2.2 ± 0.8	1.7 ± 0.7	*p* = 0.1175	1.6 ± 0.7	1.9 ± 0.7	*p* = 0.0722
Sweat rate (L/h)	0.5 ± 0.1^a^, ^b^	0.5 ± 0.1^a^, ^b^	0.4 ± 0.1	0.3 ± 0.1	0.4 ± 0.1	0.3 ± 0.1	*p* < 0.0001	0.5 ± 0.1	0.4 ± 0.1	*p* = 0.0227	0.4 ± 0.1	0.5 ± 0.1	*p* < 0.0001

*Note:* Data are presented for each compliant and noncompliant trial or combined across each compliant and noncompliant trial as mean ± SD and were analyzed using a one‐way ANOVA or *t*‐test. Exact *p* values reported where possible.

^a^ Different from Trial D (*p* < 0.05).

^b^ Different from Trial F (*p* < 0.05).

## 4 Discussion

This secondary analysis (1) examined the effectiveness of utilizing the NIOSH RAL curves depicted in NIOSH [5, Figure 8‐1] to achieve thermal equilibrium and prevent core body temperature from exceeding 38.0°C and (2) compared the core temperature responses during NIOSH RAL curve compliant and noncompliant trials of varying severity. This analysis also explored the magnitude of the discrepancy between the RAL curves and equations and its influence on core temperature responses during a simulated half‐workday in the heat. The primary finding was that in most circumstances, mean and peak core temperature were < 38.0°C and that thermal equilibrium was reached during NIOSH compliant trials. In contrast, violating the NIOSH RALs resulted in a uniform response in which all subjects did not achieve thermal equilibrium and exceeded 38.0°C regardless of severity of the exposure. Moreover, ΔRAL, which provided an understanding of both the discrepancy between the visual representation of the RAL curves versus equations and interindividual differences in *H*_prod_, was consistently identified as an independent predictor of mean and peak core temperature and core temperature slope. These are important findings for two reasons. These findings demonstrate that compliance with NIOSH‐prescribed RALs ensures that core temperature on the population level will be maintained below the accepted thresholds for excessive heat strain (i.e., ≥ 39.2°C) [8, 21]. Additionally, for a given WBGT exposure, interindividual differences in *H*_prod_, quantified here as ΔRAL due to the time‐weighted average metabolic heat production, consistently explain variability in the quantified core temperature responses.

The original purpose of the 38.0°C threshold, established by the WHO [7] more than 50 years ago, was to ensure the average worker's core temperature did not exceed 38.0°C. Notably, it is unclear if this threshold referred to peak core temperature or to the average core temperature across an entire workday. This is important as contributions to heat‐related organ injury arise from both total heat load across a given workday and the maximum core temperature experienced [18]. For example, in preclinical models of heat‐related illness, heat stroke occurs at a critical temperature (~42°C), but increasing the time under heat stress prior to heat stroke occurring elevates long‐term sequalae and end organ injury [22]. Thus, in the absence of exceptionally elevated core temperatures, mean core temperature across the workday is likely an important consideration to the 38.0°C threshold. Several previous investigations have examined the efficacy of occupational heat stress recommendations (e.g., RALs or Threshold Limit Values [TLVs]) to prevent core temperature from exceeding 38.0°C [23, 24, 25, 26]. In the studies directly examining the NIOSH heat stress recommendations peak core temperature reached 38.1°C during continuous work [26] and 37.9°C–38.1°C when work–rest is issued at 2:1 [25] based on NIOSH [5, Table 6‐2]. This largely parallels the findings presented here. Similarly, Bartman et al. [26] extrapolated their core temperature data from 2 to 4 and 8 h of exposure and reported minimal increases in core temperature above 38.0°C. This further supports the findings in the present study that most of the subjects achieved thermal equilibrium during the simulated half workday. Indeed, the average slope of the linear regression was ~0.08°C/h indicating that minimal heat gain would be anticipated with additional exposure duration. Importantly, we extend much of the previous work that has been limited to 2–3 h of exposure [23, 24, 25, 26]. Future work should examine core temperature responses during full workdays [27].

Another purpose of the present study was to examine the comparative efficacy across each of the NIOSH‐compliant trials (i.e., work–rest ratio at a given WBGT and metabolic heat production). Our findings demonstrate that the RAL curves are equally protective across a range of WBGT, work–rest ratios, and H_prod_. Previous work has shown similar findings when comparing shorter (20 min) and longer (40 min) duration work bouts with the same work–rest ratio (2:1) [25]. In contrast, Meade et al. [24] showed differences in peak core temperature during 2 h exposures that differed in work–rest ratio and WBGT, but at a fixed metabolic heat production. A 1:3 work–rest ratio implemented in a 31°C WBGT condition resulted in the lowest peak core temperature compared to 1:1 and 3:1 work–rest ratios in 30°C and 29°C, respectively. Interestingly, when Meade and colleagues projected core temperature responses from 2 h (observed) to 4 h (projected), excessive heat strain was most likely to occur in the lower WBGT but higher total work conditions. This parallels the findings in the present study, as Trial A (continuous work) resulted in the highest observed core temperatures and the highest rate of heat gain (0.14°C/h). This highlights the import consideration of metabolic heat production (or total workload) and its implications on core temperature. Indeed, previous work—especially regarding heat‐related kidney pathology—highlights that laborers with a high workload (e.g., sugarcane cutters) have elevated risk of excessive heat strain and heat‐related sequelae compared to other sugarcane harvest workers (e.g., field support, irrigation workers) exposed to the same environmental conditions [28].

Utilizing the RAL curves systematically overestimated the RAL based on the equation (~2.5°C ΔRAL) (Table 2). The multiple logistic and linear regression analyses revealed that there is an effect of ΔRAL on mean and peak core temperature, as well as the rate of heat gain (i.e., thermal compensability), which was independent of trial (Tables 3 and 4). Despite these differences, mean and peak core temperatures were generally maintained below the 38.0°C threshold. This finding supports that both the RAL curves and equations provide roughly equivalent levels of protection and that only exceeding the RAL by a larger extent is core temperature potentially excessive. Importantly, while the RAL equations do not require significant input to determine exposure limits, the visual depiction of the RAL curves may be simpler to implement in the workplace.

### 4.1 Experimental Considerations

Given that the present study was a secondary analysis to a registered clinical trial (NCT04767347) there are several experimental considerations pertaining to the previous studies [14, 15, 17] that also apply here. Most importantly, particularly for the generalizability of the findings, the compliance with NIOSH heat stress recommendations was only permitted under laboratory‐controlled environmental conditions where metabolic heat production remained constant during the 4‐h exposure. This contrasts what often occurs on the job, where during half of a typical workday fluctuations in environmental conditions and metabolic heat production variation would be anticipated [29, 30]. However, the selected work intensities (i.e., metabolic heat production) represent the average of several physical labor activities (e.g., walking, lifting, shoveling) as identified from the compendium of physical activities [31]. Moreover, taken in its entirety NCT04767347 was completed in participants that were self‐reported to be healthy and aged 20–40 years. Thus, combined with the findings of the previous study [14, 15, 17, 32], it may not be broadly generalizable to the general population of workers exposed to heat stress with ages across the entire adult lifespan [33] or who carry additional risk factors that may further exacerbate heat‐related illness during occupational heat stress (e.g., hypertension, diabetes, obesity) [32]. Finally, the present study examined the RAL for unacclimatized workers. It would be integral to examine the REL curves for acclimatized workers, particularly compared to establishing RELs with the equations. Given that the difference in RAL between the observed and calculated RAL from the equations was not related to the mean and peak core temperature responses, it could be speculated that we would observe similar findings in studies of the REL curves.

#### 4.1.1 Perspectives and Significance

There are several aspects of the present study that provide significant empirical evidence for mitigating excessive heat strain and dehydration in workers exposed to heat. First, when the work–rest ratio is a function of WBGT and metabolic heat production as prescribed by the NIOSH RAL curves, excessive heat strain is largely mitigated (i.e., mean and peak core temperature < 38.0°C). In 65/70 (93%) and 44/70 (63%) mean or peak core *T*_C_ never exceeded 38.0°C and only a single observation (out of 70 total observations across a range of WBGT conditions and metabolic heat productions) did core temperature exceed 39.0°C (~1.4%). Thus, when adhering to the NIOSH heat stress recommendations, the overall purpose is achieved. To our knowledge, this is one of the most comprehensive examinations of various combinations of environmental conditions, work rates, and work‐rest ratios. Second, we provide empirical evidence supporting ad libitum drinking for preventing dehydration when fluids (i.e., up to 237 mL of cool sports drink) are readily available to the subject. This challenges long‐standing conjecture that ad libitum drinking would result in inadvertent dehydration due to insufficient replacement of fluid loss via sweating [34]. Sweat rates across the NIOSH complaint trials were relatively modest (~0.3–0.5 L/h) reducing the need for large volumes of fluid to be replaced. That stated, we still observed sufficient ad libitum drinking in noncompliant conditions even when all subjects exceeded excessive heat strain (i.e., in Trial H). Third, despite the difference in RALs when determined by the curves, the data from the present study render the curves a viable option for workers and employers to determine and issue work–rest guidance based on the environmental conditions (i.e., WBGT) and work intensity (i.e., metabolic heat production). This is important as the RAL equations require the determination of the time‐weighted average metabolic heat production, and Tables 6‐2 and 6‐3 in the NIOSH [5] heat stress recommendations offer guidance on heat index and categorical work intensities (e.g., low, moderate, or heavy). Thus, the primary conclusion is that adhering to the NIOSH heat stress recommendations (from NIOSH [5, Figure 8‐1]) largely prevents core temperature from exceeding 38.0°C, despite an inadvertent difference from the RAL at any given WBGT and metabolic rate. This is consistent with their original purpose of preventing at least 95% of individuals from the risk of heat‐related illnesses.

## Author Contributions

Drs. Hess, Johnson, Hostler, and Schlader conceived and designed the work; Drs. Hess and Schlader and Ms. Hite, Ms. Heikkinen, Ms. Tarr, and Ms. Tourula contributed to the acquisition, analysis, and/or interpretation of data for the work; Drs. Hess, Johnson, Hostler, and Schlader drafted, edited, and revised the manuscript for important intellectual content. All authors approved the final version to be published; and all authors state their agreement to be accountable for all aspects of the work in ensuring that questions related to the accuracy or integrity of any part of the work are appropriately investigated and resolved.

## Disclosure

This article's contents are solely the responsibility of the authors and do not necessarily represent the official views of the National Institute for Occupational Safety and Health.

## Ethics Statement

The studies were approved by the Institutional Review Board at Indiana University (IRB# 1902420140), conformed to the Declaration of Helsinki, and were registered at clinicaltrials.gov (NCT04767347).

## Conflicts of Interest

The authors declare no conflicts of interest.
